# Biliverdin Reductase inhibitors did not improve severe unconjugated hyperbilirubinemia *in vivo*

**DOI:** 10.1038/s41598-017-01602-w

**Published:** 2017-05-10

**Authors:** Remco van Dijk, Sem J. Aronson, Dirk R. de Waart, Stan F. van de Graaf, Suzanne Duijst, Jurgen Seppen, Ronald Oude Elferink, Ulrich Beuers, Piter J. Bosma

**Affiliations:** Tytgat Institute for Liver and Intestinal Research & Department of Gastroenterology and Hepatology, Academic Medical Center, University of Amsterdam, Amsterdam, The Netherlands

## Abstract

We aimed to identify potent biliverdin reductase (BVRA) inhibitors as a novel concept for the treatment of severe unconjugated hyperbilirubinemia. 1280 FDA-approved compounds were screened *in vitro* for their ability to inhibit human and rat BVRA activity ﻿and 2﻿6 compounds were identified as BVRA inhibitors﻿. Montelukast and Disulfiram were sel﻿ected as potentially clinically applicable drug﻿s and tested to reduce serum unconjugated bilirubin (UCB) levels in the Ugt1a1-deficient rat, a model for chronic unconjugated hyperbilirubinemia. Oral administration of Disulfiram was toxic in the Ugt1a1-deficient rat (weight loss, transaminase elevation). Oral Montelukast administration led to low serum concentrations and did not alter serum UCB levels. Intraperitoneal injections of Mont﻿elukast resulted in concentrations up to 110 μmol/L in serum and 400 μmol/L in the liver. Still, serum UCB levels remained unaltered. This first study on biliverdin reductase inhibition as a novel concept for treatment of unconjugated hyperbilirubinemia identified putative *in vitro* BVRA inhibitors. Montelukast, the clinically most suitable inhibitor, did not result in reduction of serum UCB in the Ugt1a1-deficient rat. The proposed treatment strategy will not result in amelioration of severe unconjugated hyperbilirubinemia in humans without the identification or development of more potent BVRA inhibitors.

## Introduction

Severe unconjugated hyperbilirubinemia is a critical condition which can lead to irreversible brain damage (kernicterus) already in early childhood and eventually can be lethal when left untreated^1^. Accumulation of unconjugated bilirubin (UCB) results from an imbalance between UCB production and elimination and can be caused by several pathological conditions such as extensive hemolysis or severe impaired bilirubin glucuronidation known as Crigler-Najjar syndrome^2, 3^.

**Figure 1:**
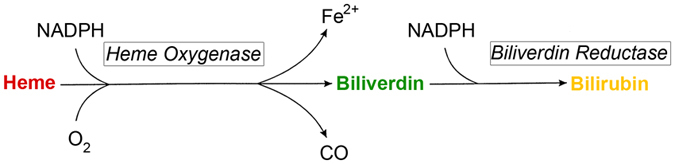
Schematic representation of the conversion of Heme into Bilirubin. Heme is catabolised into biliverdin by the enzyme Heme Oxygenase, releasing carbon monoxide (CO) and free iron (Fe^2+^). Biliverdin is further reduced by Biliverdin Reductase into unconjugated bilirubin. Both enzymes depend on nicotinamide adenine dinucleotide phosphate (NADPH) as reducing agent.

Bilirubin is the final product of heme catabolism. Old erythrocytes, which are degraded in splenocytes and liver sinusoidal cells, and cytochrome P_450_^4^, are the main source of heme production. Heme is catabolized into equimolar amounts of carbon monoxide (CO), free iron (Fe^2+^) and biliverdin by the enzyme heme oxygenase (HO)^5^. Iron is recycled for the production of heme, CO functions as a local signaling molecule and biliverdin is further reduced by the cytosolic enzyme biliverdin reductase (BVRA) into UCB (Fig. 1). The lipophilic and toxic UCB can be detoxified in the hepatocyte via conjugation with one or two glucuronide groups by the enzyme UDP-glucuronosyl transferase 1A1 (UGT1A1)^6^. Glucuronidation will render bilirubin hydrophilic, an essential modification for its excretion into bile over the apical membrane via the multidrug resistance-associated protein 2 (*ABCC2*/MRP2) transporter^7^. Under physiological conditions serum total bilirubin concentration is less than 17 μmol/L (1 mg/dL).

Newborns are likely to develop increased unconjugated bilirubinemia with total bilirubin levels above 17 μmol/L (1 mg/dL). However, only a small group will develop severe unconjugated hyperbilirubinemia, with levels above 425 μmol/L (25 mg/dL), requiring treatment. Severe neonatal jaundice is in general caused by a combination of increased bilirubin production, due to extensive breakdown of fetal hemoglobin and a shorter life span of erythrocytes, and a reduced clearance of bilirubin due to low UGT1A1 expression in the newborn liver^8^. Also other pathological conditions such as isoimmune-mediated hemolysis (e.g. Rh(D) incompatibility), erythrocyte enzymatic defects (e.g. hereditary spherocytosis and heterozygous beta-thalassemia) or inherited erythrocyte membrane defects (e.g. glucose-6-phosphate dehydrogenase deficiency) can cause severe neonatal hyperbilirubinemia^9^. In neonatal jaundice, phototherapy is the first choice of treatment. When phototherapy is not effective or infants show signs of bilirubin-induced neurologic dysfunction, treatment via exchange transfusion can be considered^10^.

Crigler-Najjar syndrome (CNS) is an autosomal recessive disorder characterized by severe unconjugated hyperbilirubinemia caused by a deficiency of bilirubin glucuronidation, due to impaired or complete lack of UGT1A1 function^11^. Phototherapy is the first line treatment for CNS^12, 13^. It is based on the formation of hydrophilic UCB photo-isomers in the skin upon exposure to light that can readily be excreted via bile or urine. Drawbacks of phototherapy are the long exposure time (up to 14 hours daily), and reduced efficacy over time. Liver transplantation, both orthotopic and auxiliary, is currently the only curative therapy for CNS^12, 13^. Due to the risk for complications and mortality, the need of lifelong immunosuppression and the long waiting list, the use of liver transplantation in CNS has its limitations.

In both disease entities described above, all treatment strategies (phototherapy, exchange transfusion and liver transplantation) are directed to eliminate the toxic UCB from the systemic circulation^14^. Rather than inducing bilirubin elimination, inhibition of bilirubin production is another potential strategy for decreasing serum UCB levels. This principle was first proven to be effective by the use of synthetic metalloporphyrins that inhibit the breakdown of heme into biliverdin by competitive binding to HO^15^. Small clinical trials showed a beneficial effect of metalloporphyrins in jaundiced neonates by lowering the maximum serum bilirubin concentration, lower frequency of severe hyperbilirubinemia, decrease the need for phototherapy, and shorter duration of hospitalization^16^. Unfortunately no reports were made on the amount and severity of neurotoxicity, death and long term neurological outcome of these patients. Although these clinical trials looked very promising, the use of metalloporphyrins has never been registered due to its severe side effects, such as photo-sensitivity of the skin, diminished CYP_450_ activity and iron deficiency anemia^17^. Metalloporphyrins also induce HO expression^17^, and will therefore not be suitable for long term use, as required in CNS.

Inhibition of the downstream step in the generation of bilirubin, the conversion of biliverdin to bilirubin, has not yet been investigated as a potential treatment for severe unconjugated hyperbilirubinemia. This step is catalyzed by biliverdin reductase (BVRA). Inhibition of this enzyme will reduce bilirubin production without affecting heme catabolism, resulting in biliverdin as the end product. Biliverdin is a hydrophilic, non-toxic compound which can be excreted in both bile and urine without any further conjugation steps^18^. Conversion of biliverdin to bilirubin is not seen in all species. Most bird and fish species completely lack biliverdin reductase and excrete exclusively biliverdin via their feces^19, 20^. Although bilirubin has been recognized as an anti-oxidant^21, 22^ and shows to have anti-inflammatory properties^23, 24^, the necessity for the conversion of biliverdin to bilirubin still forms an enigma. The identification of two adult patients with complete lack of BVRA activity due to a nonsense mutation in the BVRA gene, revealed that it is compatible with life and does not result in disease^25^. This indicates that finding an effective, non-toxic BVRA inhibitor could be an option to treat severe unconjugated hyperbilirubinemia without major adverse effects.

Aiming to treat severe unconjugated hyperbilirubinemia by decreasing bilirubin production, we developed a screen for potential BVRA inhibitors. A semi-high-throughput assay was set up to determine BVRA activity after which 1280 FDA/EMA approved compounds were screened for their ability to inhibit BVRA. Two inhibitors that could potentially be applied in a clinical setting were selected for subsequent testing *in vivo* in the Ugt1a1-deficient Gunn rat, an animal model for inherited severe unconjugated hyperbilirubinemia.

## Materials and Methods

### Cloning of rat and human biliverdin reductase A

Reverse transcribed RNA isolated from rat and human liver was amplified with Phusion high-fidelity Polymerase (New England Biolabs Inc, Ipswich, MA, USA) using the primers listed in Supplementary Table 1, and upon incubation with Taq Polymerase amplicons were cloned into a TA-cloning vector (Sigma-Aldrich, Steinheim, Germany) and sequenced completely using BigDye Terminator v1.1 (Life technologies, Carlsbad, USA). Both BVRA cDNAs were inserted into a lentiviral vector behind a constitutive CMV promoter (pLV.CMV.bc.Puro) and lentivirus was produced in HEK293T cells using transient transfection^26^.

### Production of rBVRA and hBVRA containing cell lysates

HEK293T cells were seeded in a T162 cm² flask and upon reaching 30–40% confluence lentivirus containing the hBVRA or rBVRA transgene was added in the presence of 10 μg/ml DEAE Dextran. Four hours thereafter, the medium was refreshed with complete DMEM. 48 hours after transduction the cells were washed and harvested in ice-cold PBS. Cells were then pelleted at 1500 rpm at 4 °C for 10 minutes and taken up in a hypertonic buffer (10 mM HEPES, 40 mM KCl, 2 mM MgCl_2_, 10% gycerol). After 15 minutes on ice, 25 stokes with a dounce homogenizer were used to break the cells. To remove cell membranes and debris, the homogenate was centrifuged at 80.000 RCF (xg) for 1 hour at 4 °C and the supernatant was harvested, aliquoted and stored at −80 °C.

### Biliverdin Reductase assay and semi-high-throughput drug screen

**Figure 2:**
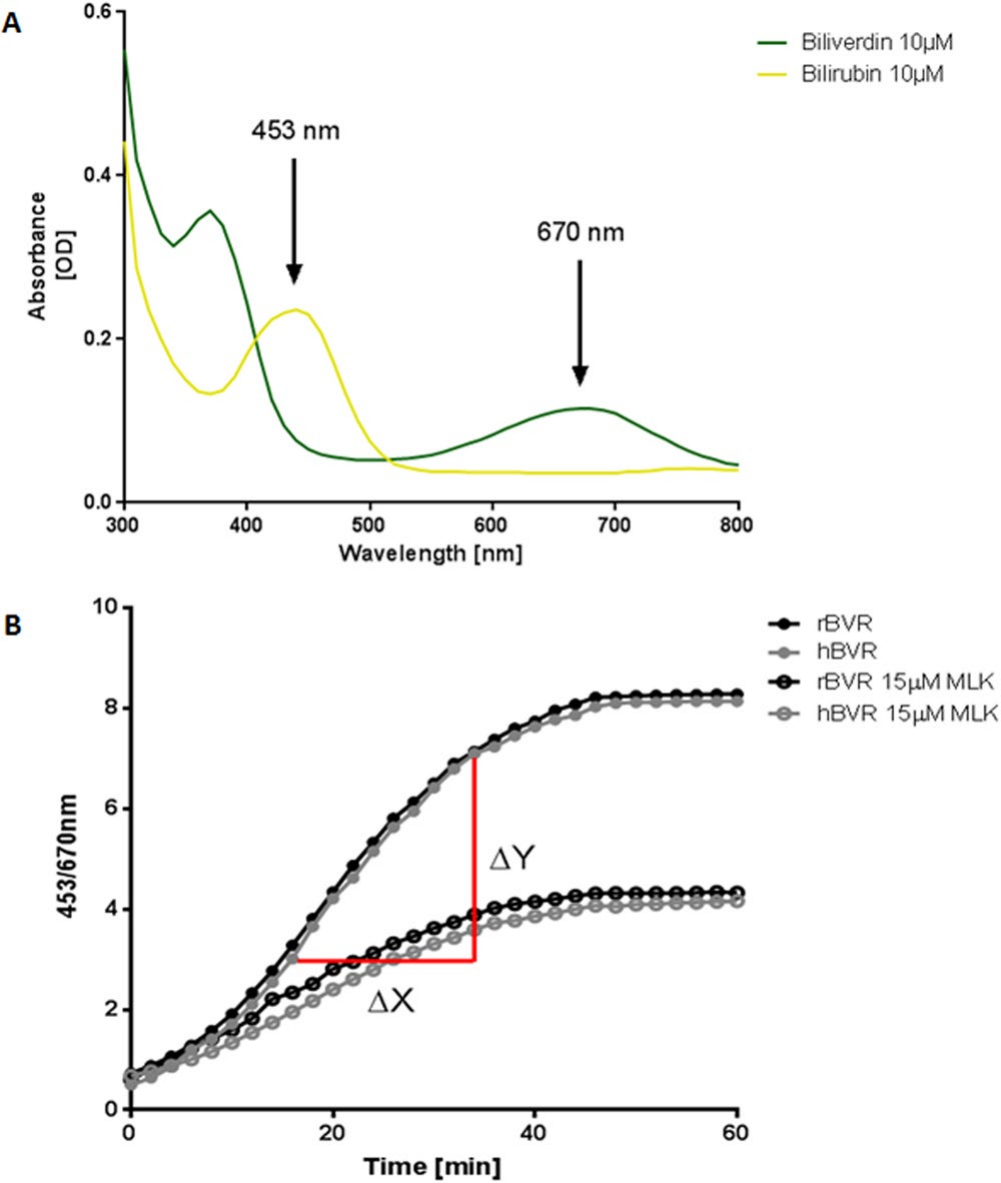
(**A**) The biliverdin reductase activity assay makes use of the different wavelengths at which bilirubin (453 nm) and biliverdin (670 nm) have their peak absorbance [OD]. The absorbance spectrum was measured at a concentration of 10 μmol/L in a total volume of 200 μL at 37 °C by a Synergy HT multi-detection reader. (**B**) Biliverdin reductase activity is derived from the speed at which biliverdin is converted to bilirubin by calculating the slope over the linear part of the enzymatic reaction. Cytosolic rat or human biliverdin reductase (rBVRA or hBVRA) was pre-incubated at 37 °C with 10 μmol/L biliverdin, bovine serum albumin (BSA 400 μg/ml) and 50 mM TrisHCL pH8.7. The reaction was started by adding NADPH to a final concentration of 100 μmol/L in a total volume of 200 μL. The conversion of biliverdin to bilirubin was determined by measuring absorbance every 2 minutes for 60 minutes and expressed as the ratio 453/670 nm. When 15 μM Montelukast (MLK) is added to the reaction, the reduced slope (∆Y/∆X) indicates reduced BVRA activity.

The biliverdin reductase activity assay was conducted in a 96-wells format in a total volume of 200 μL at 37 °C. HEK293T cytosol (5 μg total protein) was pre-incubated at 37 °C with 10 μmol/L biliverdin (Sigma-Aldrich, Steinheim, Germany), BSA (400 μg/ml) and 50 mM TrisHCL pH8.7. After 5 minutes the reaction was started by adding NADPH to a final concentration of 100 μmol/L. The conversion of biliverdin to bilirubin was determined by measuring absorbance at 453 nm (bilirubin) and 670 nm (biliverdin) every 2 minutes for 60 minutes until the plateau phase was reached using a Synergy HT multi-detection reader (BioTek Instruments Inc, Winooski, VT, USA). BVRA activity was determined by calculating the slope over the linear part of the reaction, expressed as the ratio 453/670 nm per minute (Fig. 2A,B).

A library containing 1280 FDA and EMA approved small molecules was purchased from Prestwick Chemical (Illkirch-Graffenstaden, France). The effect of 10 μM of each compound, dissolved in DMSO (Sigma-Aldrich, Steinheim, Germany) on biliverdin reductase activity was tested in duplo in two independent assays. The quality and reproducibility of this semi-high-throughput drug screen was assured with a Z-factor of >0.5 at all performed assays.

### Animal experiments

All animal experiments were performed in accordance with the guidelines and with approval of the Animal Ethics Committee of the Academic Medical Center of Amsterdam. Male Gunn rats, strain RHA j/j, from our breeding colony, were fed ad libitum and were randomly assigned to different treatment groups. Blood samples were taken by tail or saphenous vein puncture and collected in lithium heparin tubes after which plasma was separated by centrifugation. Bile samples were collected by bile duct cannulation after distal ligation of the bile duct at the time of sacrifice. Serum and bile samples were protected from light during sampling and analysis and stored at −80°C.

### Oral drug administration

Ascending doses of Disulfiram (90, 180, or 360 mg/kg/day), Montelukast (10, 30, or 90 mg/kg/day) in 1% DMSO or vehicle control were administered to male Gunn rats (12–16 weeks old) over a period of 3 weeks via oral gavage.

### Intraperitoneal drug administration

Male Gunn rats weighing 30–50 grams (4 weeks of age) were administered 300 mg/kg/day Montelukast in Hanks’ Balanced Salt solution (HBSS) buffered with 15 mM HEPES to pH 8.0 or vehicle control by bi-daily injection (150 mg/kg per injection) in the peritoneal cavity using a 23 gauge needle for 2, 5 days. In total each rat received 5 i.p. injections.

### Bilirubin and biliverdin measurements

Serum total and direct bilirubin were determined with standardized diazo assay by the Department of Clinical Chemistry of the hospital. For High-performance liquid chromatography (HPLC) analysis of both bilirubin fractions and biliverdin in serum, urine and bile the protocol was adapted from the method described by Spivak and Carey^27^. In brief, urine and plasma samples were de-proteinized with 2 volumes of methanol. Following centrifugation for 2 minutes at 14000 rpm at 4 °C, 100 μl of the supernatant was applied to a Pursuit C18, 5 μm, 10 cm HPLC column (Varian, Middelburg, The Netherlands). Starting eluent consisted of 40% MeOH/ 60% ammonium acetate (1%, pH4.5), followed by a linear gradient to 100% MeOH in 15 minutes. Detection of biliverdin and bilirubin was performed at 390 and 450 nm, respectively. Quantification of biliverdin was done by using a calibration curve of biliverdin (Sigma-Aldrich, Steinheim, Germany). Quantification of bilirubin and conjugates was executed by using a calibration curve of unconjugated bilirubin (Sigma-Aldrich, Steinheim, Germany).

### Montelukast measurements

Serum Montelukast concentration was measured using the above described HPLC protocol with a starting eluent consisting of 50% acetonitrile/50% 20 mmol/L ammonium acetate buffer, pH 5.5, followed by a linear gradient to 100% acetonitrile. Peaks were detected at 345 nm of the UV spectrum. Quantification of Montelukast was executed by using a calibration curve of Montelukast.

### Tissue analysis

Tissue (liver, spleen and kidney) was snap-frozen in liquid nitrogen and stored at −80°C. Homogenates were made in 100 mmol/L Tris-HCL pH 7, 8 buffer and bilirubin, biliverdin and Montelukast concentrations were measured by HPLC.

### Statistical analysis

To determine significance of BVRA inhibition *in vitro* between different concentrations a one way ANOVA with Bonferroni’s correction was used. A student’s t test was used to calculate differences in serum bilirubin concentrations between the treated and vehicle group.

## Results

### Biliverdin reductase inhibitor *in vitro* drug library screen

**Figure 3:**
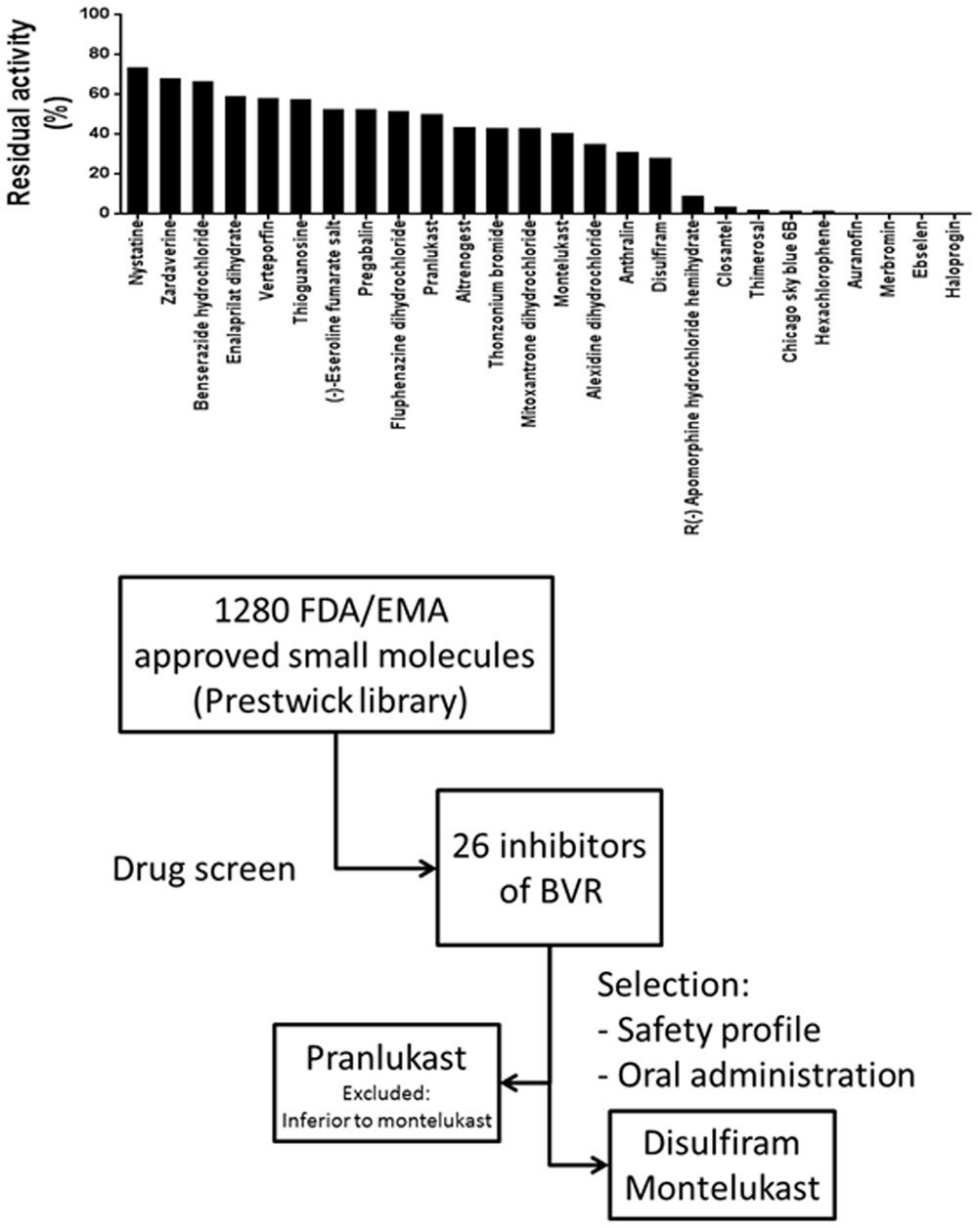
(**A**) 26 compounds were identified to inhibit human Biliverdin Reductase at a concentration of 10 μmol/l during a semi-high-throughput screen of a drug library containing 1280 FDA and EMA approved small molecules. (**B**) Two compounds were selected for *in vivo* studies after consideration of safety profile for long-term dosing and possibility of oral administration.

After establishing a stable and highly reproducible BVRA-activity assay (Fig. 2A) a drug library containing 1280 FDA and EMA approved small molecules (Prestwick Chemical) was tested for their ability to inhibit hBVRA activity *in vitro*. 26 compounds were found to inhibit hBVRA activity between 34 to 99% at the used concentration of 10 μmol/L (Fig. 3A). Of those potential candidates, those that can be administered orally and can be given long-term without potential toxic effect in the clinical setting were selected. Based on these criteria, two potential candidates for further dose response testing remained (Fig. 3B).

The leukotriene receptor antagonist Montelukast (Singulair), which is used as a complementary therapy for asthma, reduced BVRA activity by 60% during the drug library screen. A second leukotriene receptor antagonist, Pranlukast (Onon), also showed capacity to reduce BVRA activity by 50% during the drug library screen. Because Montelukast showed more potency, Pranlukast was left out for further studies (Fig. 3A).

The second compound selected was the irreversible acetaldehyde dehydrogenase inhibitor Disulfiram (Antabus) which is used to support the treatment of chronic alcoholism. Disulfiram reduced BVRA activity by 75% in the semi high-through put screen (Fig. 3A).

### Montelukast and Disulfiram BVRA activity inhibition *in vitro*

**Figure 4:**
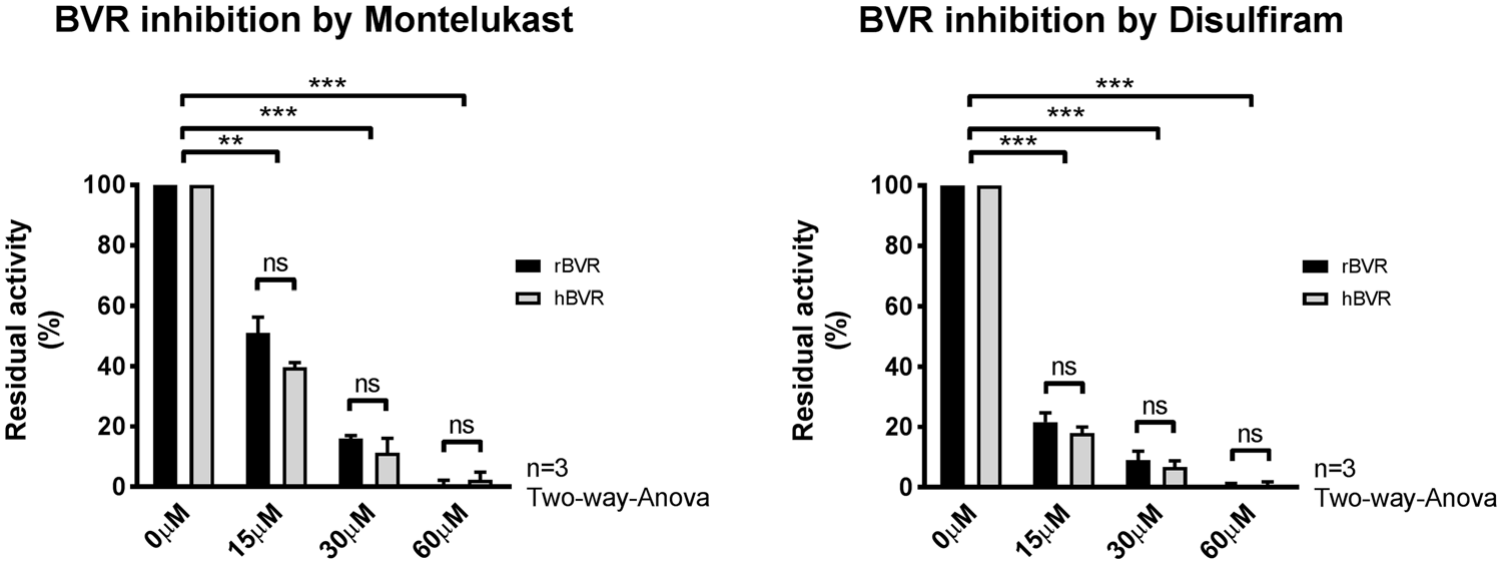
(**A**) Comparison between rat and human Biliverdin Reductase (rBVRA and hBVRA) activity in the presence of 0, 15, 30 or 60 μM Montelukast or (**B**) 0, 15, 30 or 60 μM Disulfiram. The conversion of biliverdin to bilirubin was determined by measuring absorbance over a period of 60 minutes at wavelengths 453 nm (bilirubin) and 670 nm (biliverdin). Biliverdin reductase activity was determined by calculating the slope over the linear part of the reaction and is expressed as residual activity relative to the vehicle control 1% DMSO. Data represent the mean ± SD of the data obtained from three independent experiments (n = 3). Two-way analysis of variance (ANOVA) was used for statistical analysis, **p < 0.01, ***p < 0.001.

Montelukast and Disulfiram were tested for their ability to inhibit both human and rat BVRA in four different concentrations *in vitro*. Indeed both compounds showed a dose-response effect on BVRA activity. As observed during the drug library screen, both compounds reduced BVRA activity with more than 50% with concentrations as low as 15 μmol/L. At a concentration of 30 μmol/L Montlelukast inhibited hBVRA and rBVRA by 75%. Disulfiram was even more effective and showed almost complete BVRA inhibition at concentrations above 30 μmol/L (Fig. 4A,B). Because of these positive findings both Disulfiram and Montelukast were tested *in vivo* for their ability to reduce serum bilirubin in the chronic hyperbilirubinemic Gunn rats.

### Disulfiram is hepatotoxic in the Gunn rat

Adult male Gunn rats were administered Disulfiram via oral gavage in a weekly increasing dose starting from 90 mg/kg/day increasing 2 fold every week. During the first week the rats started to lose weight in comparison to the vehicle control group. This weight loss progressed during the second week when the dose of Disulfiram was increased to 180 mg/kg/day as per protocol (Supplementary Figure 1a). Besides losing weight the animals also showed a rise in both serum bilirubin and liver enzyme (ALT) levels (Supplementary Figure 1b,c), indicating a hepatotoxic effect of Disulfiram in Gunn rats. The experiment was terminated after the second week, due to the progressive weight loss of the animals. Because of the (hepato-)toxic profile no further *in vivo* experiments involving Disulfiram were conducted.

### No effective BVRA inhibition upon oral administration of Montelukast

Adult male Gunn rats received Montelukast via oral gavage at a starting dose of 10 mg/kg/day and increasing 3-fold every week. In contrast to Disulfiram, Montelukast was well tolerated by the Gunn rat. However, even the highest dose of Montelukast (90 mg/kg/day) did not alter serum bilirubin levels (Supplementary Figure 2a). To investigate if serum Montelukast concentrations were in the range for potential rBVRA inhibition trough levels were determined using HPLC. This revealed serum Montelukast trough levels were only between 1 and 8 μmol/l, clearly below the concentration needed to inhibit rBVRA (Supplementary Figure 2b).

### High serum Montelukast concentrations do not result in reduced serum bilirubin in young Gunn rats

**Figure 5:**
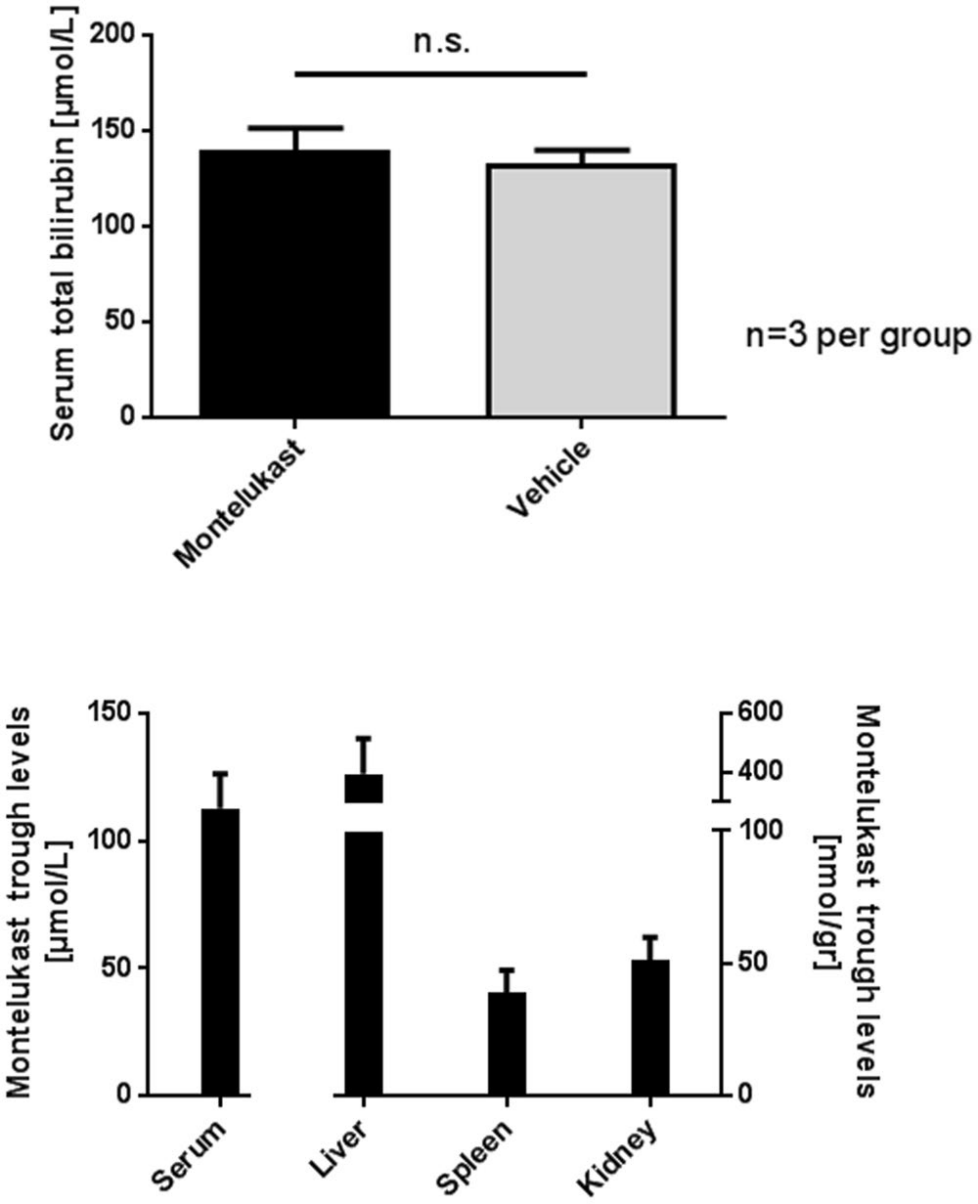
(**A**) Total serum bilirubin is not decreased in 4 weeks old male Gunn rats after bi-daily intraperitoneal injection of 150 mg/kg Montelukast for 2, 5 days (5 injections), compared to animals receiving vehicle control. Data represent the mean ± SD of three animals per group. (**B**) Concentration of Montelukast in serum, liver, spleen and kidney of 4 weeks old male Gunn rats after bi-daily intraperitoneal injection of 150 mg/kg Montelukast for 2, 5 days (5 injections). Animals were sacrificed 5 hours after last Montelukast administration. Tissue homogenates were made in 100 mmol/L Tris-HCL pH 7, 8 buffer and concentration of Montelukast was measured by HPLC. Data represent the mean ± SD of three animals per group.

To achieve higher *in vivo* concentrations, young male Gunn rats were injected intraperitoneally with Montelukast. For three days they received bi-daily injections (150 mg/kg). This indeed resulted in high serum Montelukast concentrations up to 112.0 ± 27.0 μmol/L. The Montelukast concentration was variable in different tissues ranging from 38.7 ± 14.9 nmol/gram in the spleen to 51.1 ± 15.0 nmol/gram in the kidney and accumulating in the liver to 396.0 ± 168 nmol/gram (Fig. 5B). Although these concentrations would strongly inhibit BVRA activity *in vitro*, no effect was observed on the serum bilirubin concentrations in comparison to the vehicle treated group (Fig. 5A). Furthermore, no biliverdin was detectable in serum, urine, liver and spleen (data no shown), suggesting the inhibition of rBVRA, if any, was insufficient.

## Discussion

In the present study, we have demonstrated that biliverdin reductase (BVRA) activity can be successfully inhibited *in vitro* by at least 26 FDA/EMA approved compounds, including the irreversible acetaldehyde dehydrogenase inhibitor Disulfiram and the leukotriene receptor antagonist Montelukast. However, *in vivo* administration of Montelukast did not result in a decrease of serum bilirubin, while Disulfiram appeared to be hepatotoxic to the Ugt1A1-deficient Gunn rat, the animal model for hereditary unconjugated hyperbilirubinemia. Both compounds therefore appeared not suitable to treat severe unconjugated hyperbilirubinemia in this animal model.

Both neonatal jaundice and Crigler-Najjar syndrome, marked by highly elevated serum levels of unconjugated bilirubin, can cause severe central nervous damage with life-long disabilities and can even be lethal when left untreated. Current established treatments are based on the elimination of UCB, rather than on reducing the production of UCB. Based on two subjects described in literature lacking biliverdin reductase activity that did not express a disease phenotype^25^, we postulated that inhibition of this enzyme is an interesting new target for the treatment of severe unconjugated hyperbilirubinemia. Two out of the 26 FDA approved compounds that showed to inhibit BRVA had a favorable safety profile for potential clinical application in the treatment of CNS.

Disulfiram has been used for more than 60 years in treatment of alcohol dependence. By inhibiting the enzyme acetaldehyde dehydrogenase an accumulation of acetaldehyde will occur after alcohol intake, resulting in a disulfiram-ethanol reaction with symptoms including: flushing of the skin, accelerated heart rate, shortness of breath, nausea, vomiting, throbbing headache, postural syncope, and circulatory collapse^28^. Disulfiram is also known to inhibit cytochrome P_450_ 2E1 and by forming complexes with metals it can function as a protease inhibitor^29^. In this study we found for the first time that Disulfiram also acts as a biliverdin reductase inhibitor. *In vitro* Disulfiram showed to be a very effective BVRA inhibitor with a concentration of only 10 μmol/L resulting in a 75% activity reduction. However, *in vivo* oral administration of Disulfiram resulted in severe weight loss and possible hepatotoxicity in male Gunn rats. Several clinical studies have shown that the use of Disulfiram can lead to liver tests abnormalities in 25% of the patients of whom 10–16% suffered from severe hepatotoxicity resulting in liver transplantation or death^30^. Although the underling mechanism is not fully understood yet, it is thought that different processes such as hypersensitivity and direct toxic effects of Disulfiram metabolites may play a role^30, 31^. A large proportion of Disulfiram is eliminated via bile after glucuronidation of its metabolite diethyldithiocarbamate^32^. Absence of this detoxification mechanism could in part explain the hepatotoxicity of disulfiram in our Ugt1a1 deficient animal model, rendering this drug unsuitable for the use in patients with CNS. Further evaluation of the pharmacokinetics of disulfiram is required. However in this animal model, it is challenging since methods to accurately measure disulfiram and its metabolites in serum are currently lacking^33, 34^.

Montelukast has also shown to be a potent *in vitro* inhibitor of both human and rat BVRA. Montelukast is a cysteinyl leukotriene receptor type 1 antagonist which is clinically used as add-on therapy to inhaled corticosteroids for asthma patients^35^. By blocking the leukotriene pathway it improves symptoms of asthma and reduces inflammation^36^. Montelukast is generally well tolerated in both pediatric and adult patients, with few adverse reactions reported^35^. This lipophilic compound with high protein binding affinity^37^ could possibly displace UCB from albumin by competitive binding, although literature to support this assumption lacks. No effect was seen on the bilirubin metabolism in the Gunn rat after both oral and intraperitoneal administration. Montelukast is a very effective cysteinyl leukotriene receptor type 1 antagonist with only low nanomolar concentrations needed to achieve clinical effects^38^. Although a much higher oral dose was used (90 mg/kg/day vs 0.125 mg/kg/day), serum concentrations reached a maximum of only 4 μmol/L in the Gunn rat. As observed in the *in vitro* studies, a concentration above 60 μmol/L is most likely necessary to completely inhibit BVRA and to result in a clinical effect on the bilirubin metabolism. We did not observe any alteration in the serum bilirubin levels in Gunn rats treated with oral Montelukast, which indeed could be caused by inadequate serum concentrations of the drug. To obtain proof of concept, younger male Gunn rats were bi-daily injected intraperitoneally for three days with Montelukast. Although this resulted in a significant increase in serum Montelukast concentrations up to 112 μmol/L and as high as 396 nmol/gram in the liver, the required concentration for complete BVRA inhibition was not reached in all tissues. Serum bilirubin levels remained unaltered and biliverdin did not appear in serum or urine during treatment. Whether complete BVRA inhibition in al tissues would lead to a decrease of serum bilirubin levels remains uncertain, but with the proposed inhibitors it is unlikely that complete BVRA inhibition *in vivo* can be achieved.

The other compounds identified as potential BVRA activity inhibitors during the semi-high-throughput screen are either not orally administrable (e.g. Anthralin and Haloprogin), used as stabilizers/preservatives (e.g. Thimerosalm) or have inferior safety profiles (e.g. Auranofin and Apomorphine) and were therefore not further investigated both *in vitro* and *in vivo*.

The reticuloendothelial system is the site of erythrocyte degradation and heme catabolism. Kupffer cells and other macrophages express large amount of HO-1 and are responsible for 50–75% of all bilirubin formation^39, 40^. Biliverdin reductase is expressed widely throughout the body, including macrophages. Measurement of Montelukast after intraperitoneal administration showed high concentrations in serum and liver, but possibly insufficient concentrations in spleen and kidney for complete BVRA inhibition. Unaltered serum bilirubin levels could indicate that only a small amount of residual BVRA activity could be sufficient to support bilirubin production, resembling the high efficiency of UGT1A1^41^. Biliverdin reductase is a cytosolic protein^42^, therefore it is important that Montelukast is taken up by the macrophages to have any effect on the bilirubin metabolism. The exact mechanism, however, by which Montelukast is taken up by cells remains to be determined^43^.

In conclusion, we report for the first time that multiple compounds have the ability to inhibit BVRA activity *in vitro*. Because of the potential clinical use in patients with severe unconjugated hyperbilirubinemia, Disulfiram and Montelukast were further investigated in the appropriate animal model. However, both compounds did not lower serum bilirubin levels *in vivo*, and Disulfiram even proved to be toxic in rats lacking Ugt1a1 activity. Unless a potent BVRA inhibitor with high bioavailability in all tissues is identified, the proposed treatment strategy will not result in amelioration of severe unconjugated hyperbilirubinemia in humans.

## Electronic supplementary material

Supplementary info
