# Supplementary material for: Biliverdin Reductase inhibitors did not improve severe unconjugated hyperbilirubinemia in vivo
Source: Sci Rep. 2017 May 10;7:1646. doi: 10.1038/s41598-017-01602-w (PMC5431759; doi:10.1038/s41598-017-01602-w)
Supplement: Supplementary file 1 — Supplementary info [file 41598_2017_1602_MOESM1_ESM.pdf]

# Biliverdin Reductase inhibitors did not improve severe unconjugated hyperbilirubinemia *in vivo*

Remco van Dijk[1*], Sem J. Aronson[1*], Dirk R. de Waart[1], Stan F van de Graaf[1], Suzanne Duijst[1], Jurgen Seppen[1], Ronald Oude Elferink[1], Ulrich Beuers[1] and Piter J. Bosma[1]

[1] Tytgat Institute for Liver and Intestinal Research & Department of Gastroenterology and Hepatology, University of Amsterdam, the Netherlands.

**Author Contributions statement:** [*] RD and SA have contributed equally to this manuscript: collecting data, performing analysis and writing manuscript. PB initiated project. RdW and SD collected data. SvdG, JS, ROE, UB and PB critically revised the manuscript. All authors approve manuscript.

**Address for correspondence:**

Dr. Piter J. Bosma

Department of Gastroenterology and Hepatology

Tytgat Institute for Liver and Intestinal Research

Academic Medical Center, S1-1768

University of Amsterdam

Meibergdreef 69

1105 BK Amsterdam

The Netherlands

Tel.: +31-20-566-8850

Fax: +31-20-566-9190

E-mail: p.j.bosma@amc.uva.nl

**Keywords:** Biliverdin Reductase; BVRA; Crigler-Najjar; Disulfiram; Montelukast; Ugt1a1-deficient rats; Unconjugated Hyperbilirubinemia

**Competing financial interests statement:** None of the authors have any conflicts of financial interest to disclose with regard to this work.

**Supplementary figures**

| Gene (exon) | Primer sequence | | Length bp |
|---|---|---|---|
| Rat BLVRA | F<br>R | TCTGTCTGTCTTCGGACACT<br>TGGTTGATAGGACCAAACCCTG | <br>977 |
| Human BLVRA | F<br>R | CGTCAGTGACCGAAGGAA<br>ATGCTGGTGCCATCTTGGA | <br>959 |

**Supplementary table 1: Primer sequences used for the cloning of rat and human biliverdin reductase A (rBVRA, hBVRA)**

**a**

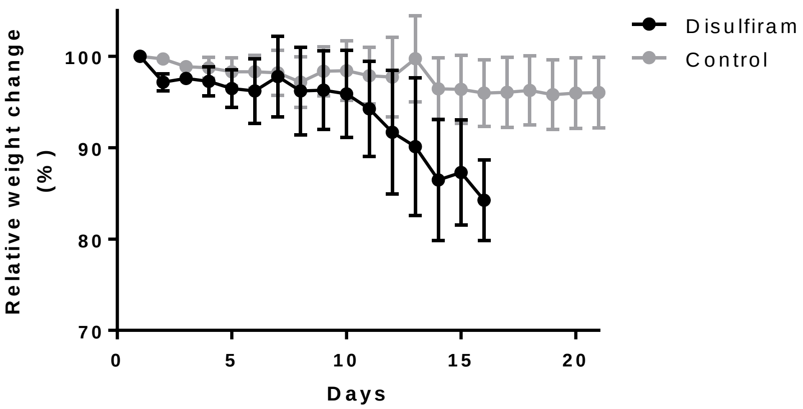


**b**

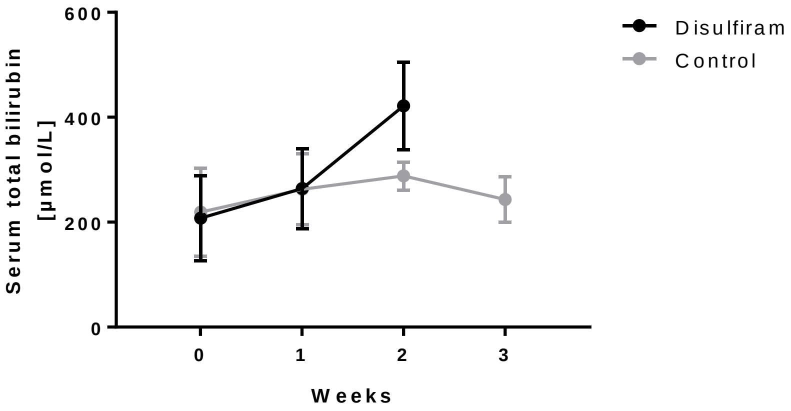


**c**

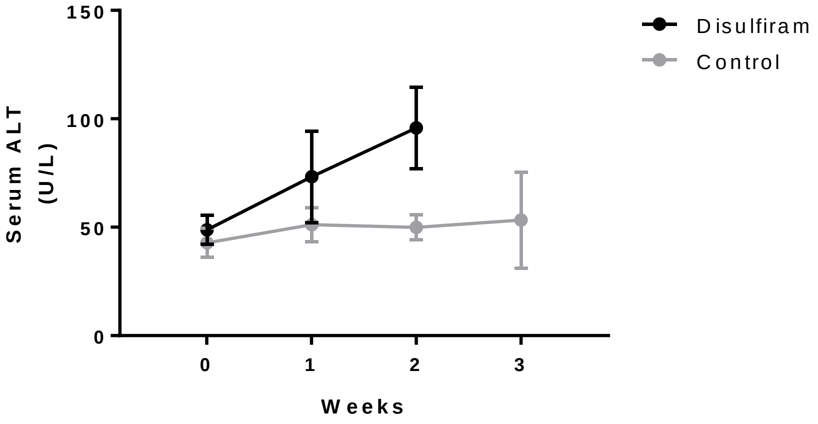


**Supplementary figure 1: Hepatotoxic effect of Disulfiram in adult Gunn rats** after 2 weeks of oral administration (90-180mg/kg/day) resulted in (a) weight loss compared to the control group (b)

increase in serum total bilirubin (µmol/L) and (c) elevation of alanine aminotransferase (ALT), leading to termination of the experiment. Data represent the mean ± SD of five animals per group.

**a**

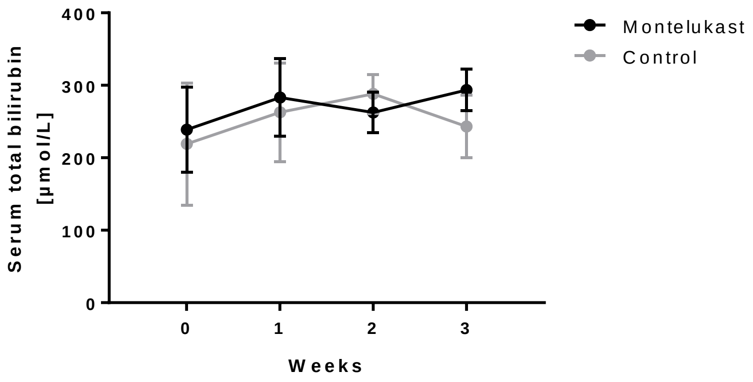


**b**

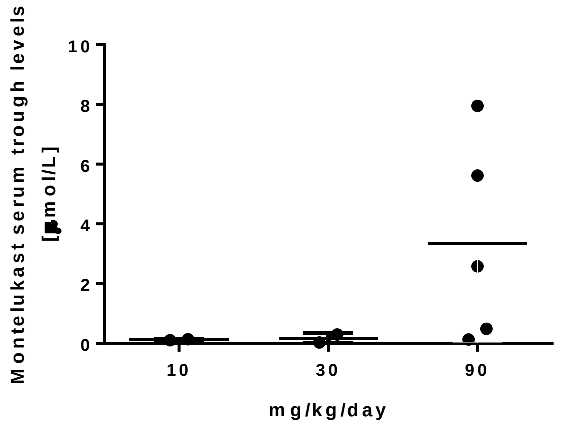


**c**

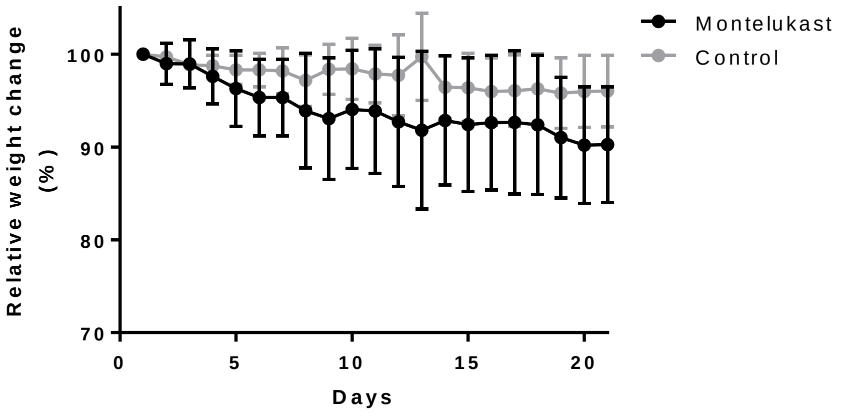

**d**

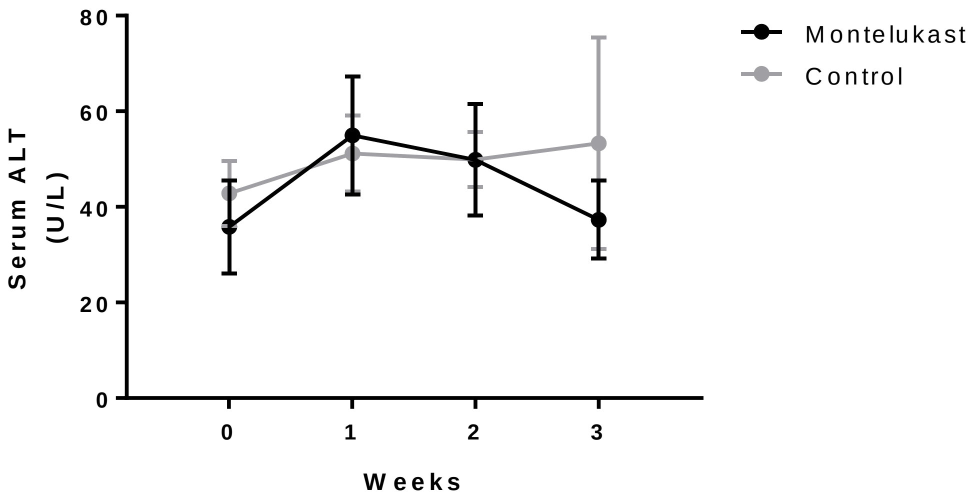


**Supplementary figure 2: Oral administration of Montelukast (up to 90 mg/kg/day)** does (a) not result in reduction of serum total bilirubin, due to (b) insufficient serum concentrations to achieve complete BVRA inhibition (>60 µmol/L). (c) no significant weight loss compared to control or (d) elevation of alanine aminotransferase (ALT) were seen in these animals. Data represent the mean ± SD of five animals per group.
